# Locally Advanced Glomus Tumor of the Stomach With Synchronous Liver Metastases: Case Report and Literature Review

**DOI:** 10.7759/cureus.51041

**Published:** 2023-12-24

**Authors:** Fabio Frosio, Carmine Petruzziello, Elia Poiasina, Michele Pisano, Alessandro Lucianetti

**Affiliations:** 1 Department of General Surgery, Azienda Socio Sanitaria Territoriale (ASST) Papa Giovanni XXIII Hospital, Bergamo, ITA

**Keywords:** case report, liver metastases, stomach, gastric tumor, glomus tumor

## Abstract

Gastric glomus tumors (GGTs) are usually rare mesenchymal neoplasms. They are typically benign, with very few metastatic cases reported and no specific guidelines on their management. Here, we present a patient with a locally advanced GGT with synchronous liver metastases. One month after resection of the GGT, emergency laparotomy was required for massive hemoperitoneum due to bleeding from the largest metastasis. Indeed, a dramatic progression of liver metastases was observed in just one month. A wide local excision is considered the treatment of choice for GGTs. In particular, this case report suggests that the resection of any liver metastases should possibly be performed at the same time as the GGT excision and not at a later stage.

## Introduction

Glomus tumors (GTs) are rare mesenchymal neoplasms arising from glomus bodies, usually occurring in the hands and feet. Gastric glomus tumors (GGTs) are exceedingly rare and are generally benign [1]. They originate from the submucosa or muscularis propria and develop as masses in the lumen or on the serosa. The most common symptoms are abdominal pain, bleeding and perforation [2]. There are no specific clinical, radiological or endoscopic features for the diagnosis. They are confirmed only by histopathology and immunohistochemistry. The presence of nuclear atypia and mitotic activity is considered indicative of malignancy. However, only a few reports of malignant GGTs have been described in the literature so far, with just a handful of metastatic cases [3].

We report an exceptional and challenging case of locally advanced GGT with synchronous liver metastases. The existing literature on metastatic GGTs has also been reviewed by consulting the MEDLINE online database through PubMed.

## Case presentation

**Figure 1:**
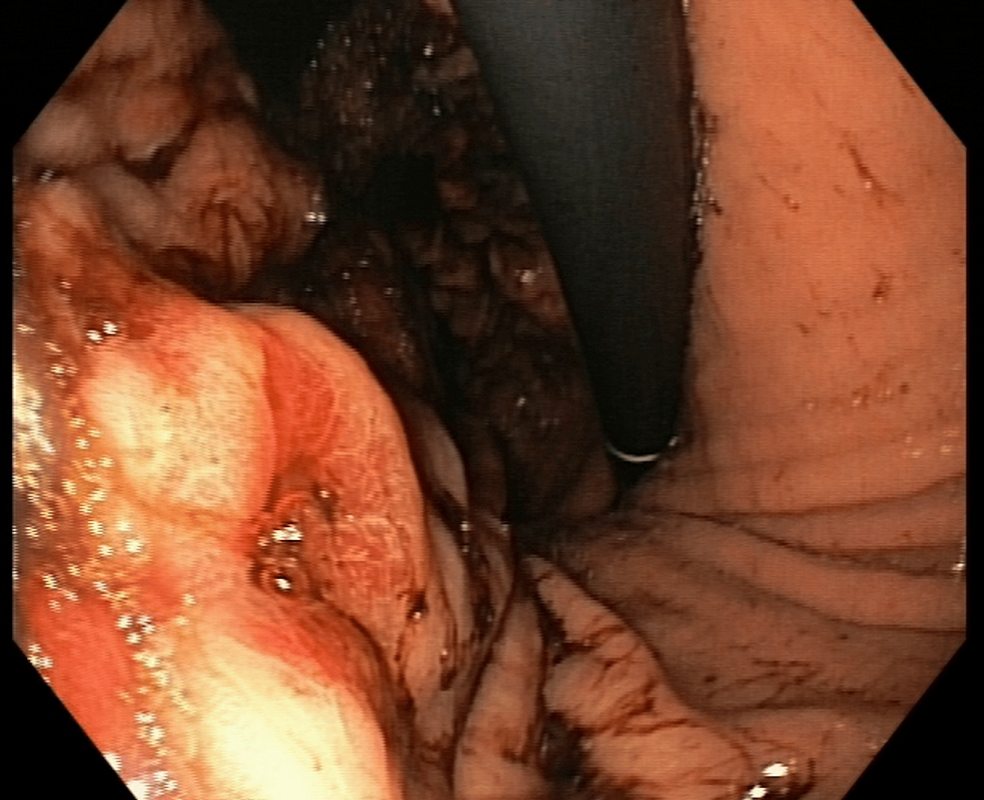
Upper gastrointestinal endoscopy: an ulcer with irregular margins is found on the body of the stomach

A 60-year-old Caucasian woman presented to the Emergency Department for worsening asthenia and dyspnea on exertion. Her past medical history included hypertension and Helicobacter pylori gastritis three years before. Vital signs were within range: blood pressure 110/70 mmHg, heart rate 90 beats per minute, body temperature 36.5 °C, oxygen saturation 98%, and respiratory rate 18 breaths per minute. There was no abdominal mass on palpation. Routine laboratory tests showed severe anemia with hemoglobin of 6.5 g/dL, requiring blood transfusion (Table 1). Upper gastrointestinal endoscopy revealed an ulcer with irregular margins on the body of the stomach over the greater curvature (Figure 1). An endoscopic biopsy was performed.

**Table 1:** Blood test results at presentation PT: prothrombin time, INR: international normalized ratio

Parameter	Results	Biological Reference Range	
Red Blood Cell Parameters			
Red Blood Cell Count	2.42	4.13 - 5.15	10^12/L
Hemoglobin	6.5	12.0 - 16.0	g/dL
Hematocrit	21.0	37.9 - 46.1	%
Mean Corpuscolar Volume	86.8	81.8 - 95.3	fL
Mean Corpuscolar Hemoglobin	26.9	27.3 - 32.2	pg
Mean Corpuscular Hemoglobin Concentration	310	310 - 360	g/L
Red Blood Cell Distribution Width	17.0	11.9 - 14.4	%
White Blood Cell Parameters			
White Blood Cell Total Count	6.07	4.2 - 9.4	10^9/L
White Blood Cell Absolute Count			
Neutrophils	4.74	2.0 - 6.7	10^9/L
Lymphocytes	0.86	1.1 - 3.4	10^9/L
Monocytes	0.36	0.2 - 0.8	10^9/L
Eosinophils	0.08	0.0 - 0.5	10^9/L
Bosophils	0.03	0.0 - 0.1	10^9/L
White Blood Cell Differential Count			
Neutrophils	78.1	40 - 80	%
Lymphocytes	14.2	20 - 40	%
Monocytes	5.9	2 - 10	%
Eosinophils	1.3	1 - 6	%
Bosophils	0.5	0.1 -2	%
Platelet Count	422	150-400	10^9/L
PT - INR	1.01	0.80 - 1.25	
Urea	43	10 - 50	mg/dL
Glucose	96	65 - 100	mg/dL
Bilirubin, Total	0.3	0.2 - 1.2	mg/dL
Protein, Total	6.0	6.0 - 8.0	g/dL
Chloride	107	98 - 107	mmol/L
Sodium	138	136 - 145	mmol/L
Potassium	4.3	3.5 - 5.0	mmol/L
Creatinine	0.86	0.3 - 1.1	mg/dL
Aspartete Aminotransferase	27	13 - 40	U/L
Alanine Aminotransferase	23	7 - 40	U/L
Pseudocholinesterase	6970	7000 - 19000	U/L
Lactate Dehydrogenase	220	120 - 246	U/L
Amylase	121	24 - 94	U/L
Lipase	71	0 - 60	U/L
C Reactive Protein	1.3	> 1.0	mg/dL

**Figure 2:**
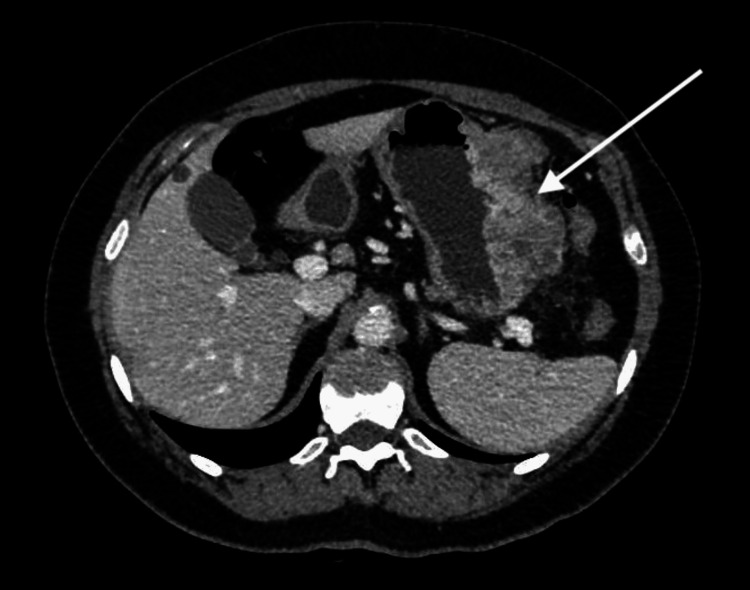
Preoperative abdominal CT scan (venous phase, axial plane): a large mass arises from the greater curve of the stomach (white arrow) CT: Computed Tomography

**Figure 3:**
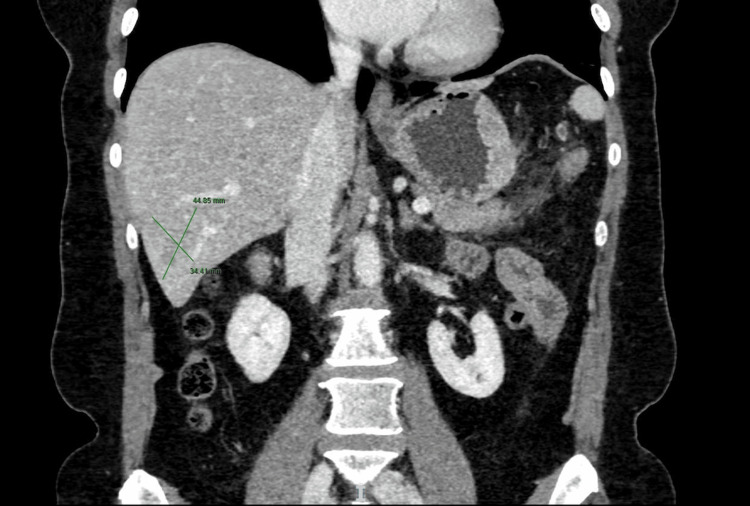
Preoperative abdominal CT scan (venous phase, coronal plane): a liver metastasis with undefined margins involves segments VI and VII CT: Computed Tomography

Abdominal computed tomography (CT) scan detected a 10 cm mass arising from the greater curve of the stomach, which displayed contrast enhancement in the arterial and venous phases (Figure 2). The mass was very close to the body of the pancreas and the transverse mesocolon, with no definite signs of infiltration. Large lymph nodes along the greater curve of the stomach were evident. CT scan also showed two liver nodules, one well-defined in segment IV and one undefined between segments VI and VII, which were consistent with metastatic lesions (Figure 3). The lesions measured 3 and at least 5 cm in diameter respectively. Also, there were pulmonary nodules bilaterally, which were too small for further characterization.

**Figure 4:**
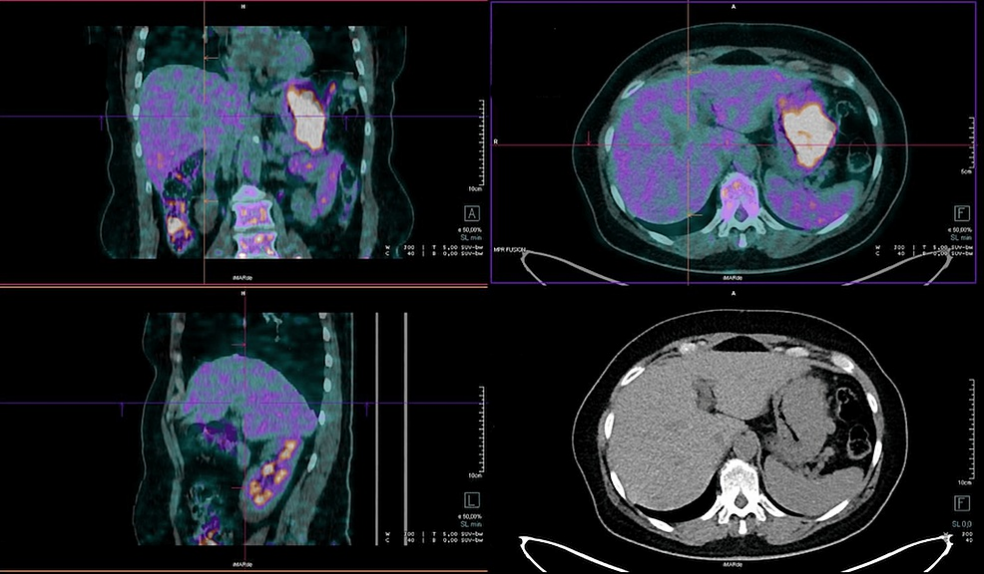
Preoperative 18-FDG PET: enhanced glucose accumulation was demonstrated on the body of the stomach, without significant radioactivity in the liver 18-FDG PET: 18-FluoroDeoxyGlucose Positron Emission Tomography

The patient was then admitted to the Oncology Unit for further workup with a stable hemoglobin above 11 gm/dL. 18-fluorodeoxyglucose (18-FDG) positron emission tomography (PET) demonstrated an enhanced radioactive glucose accumulation on the body of the stomach, with a standard uptake value (SUV) of 13.6. On the other side, no significant radioactivity was revealed in the liver or lung nodules (Figure 4).

Preliminary histological data from the endoscopic biopsy indicated a rare neoplasm, which would require more time to establish a definitive diagnosis. In this context, and given the need to determine the nature of the hepatic nodules, a percutaneous liver biopsy was performed with no further delay. The patient was subsequently discharged in good condition while awaiting the results of histological examinations.

Endoscopic biopsy revealed gastric mucosa infiltrated by medium-sized cellular elements with moderate nuclear atypia. Immunohistochemical staining showed that tumor cells were positive for vimentin and synaptophysin; LCA, CD117, EMA, SOX-1, CK7, MCK, DOG-1, CK CAM 5.2, and S100 proteins were negative. No rearrangements of SS18 and EWSR1 genes were identified on fluorescence in situ hybridization (FISH). Then, gastric carcinoma, synovial sarcoma, Ewing sarcoma, and gastro-intestinal stromal tumor were excluded as possible histotypes. Given the immunohistochemical positivity for synaptophysin, although cytokeratin was negative, a possible neuroendocrine tumor was suggested; chromogranin A level was high (21 nmol/L, normal value < 3 nmol/L), while neuron-specific enolase (NSE) was normal. Overall, these results were considered consistent with mesenchymal neoplasia. On the other hand, liver biopsy was inconclusive.

Hence, having performed a second percutaneous liver biopsy, the pathological slides were sent to the regional oncologic hub. Immunophenotype was positive for vimentin, beta-catenin, smooth muscle actin, and CD56. Ki67 was 60%. A similar pattern was identified on the liver slides. Mesenchymal neoplasia was confirmed, with only a propensity for malignant metastatic glomus tumor. Therefore, a definitive diagnosis was not established.

In the meantime, the patient had presented to the Emergency Department again for abdominal pain and melena. Further transfusions were necessary.

The case was submitted for multidisciplinary team discussion. Given the persistent bleeding and the absence of a definitive diagnosis, the panel decided on a surgical approach of the primary gastric GT with both palliative and diagnostic intent. Simultaneous treatment of liver metastases was not recommended, since it would have required the resection of at least three liver segments; hence, liver surgery would be performed at a later stage. Depending on the results of the pathological examination and possible mutations, adjuvant chemotherapy would be considered. Therefore, the patient was initially scheduled for laparoscopic wedge resection of the stomach.

During diagnostic laparoscopy, a large mass at the gastric fundus was found, at some distance from the angle of His, with a gross infiltration of the transverse colon mesentery. For this reason, we converted laparoscopic to open surgery. Liver inspection confirmed the two metastatic lesions, the largest between segments VI and VII. Finally, en-bloc wedge resection of the stomach and the transverse colon was performed; side-to-side double-layered hand-sewn colo-colonic anastomosis was made. The patient recovered uneventfully and was discharged on postoperative day eight.

Histopathological examination of the specimen revealed a 12 cm neoplasm originating from the gastric submucosa, with full-thickness infiltration of the gastric wall up to the perivisceral fatty tissue of the transverse colon. The neoplasm consisted of small monomorphic basophilic cells surrounding ectactic vascular structures and smooth muscle tissue. Wide surgical margins and lymph nodes were negative. Intraoperative peritoneal washing cytology was negative for malignancy.

With regard to immunohistochemistry, muscle-specific actin (MSA), caldesmone and synaptophysin were positive, while AE1/AE3, CAM 5.2, S100, CK7, CK20, DOG-1, NSE, and c-KIT were negative. A positive “dot-like” perinuclear staining for CD99 was detected as well. Ki67 was about 40%.

Overall, these findings were considered consistent with a gastric GT. Positivity for synaptophysin did not rule out this diagnosis, as GTs can show aberrant expression [4]. No adjuvant chemotherapy was recommended. A magnetic resonance imaging (MRI) was scheduled approximately one month after the gastric surgery, in order to proceed with liver resection early on.

Before the MRI could be performed, the patient presented to the Emergency Department on postoperative day 30 for asthenia and abdominal pain. The patient had severe hypotension and tachycardia; on examination, she had a distended and tense abdomen. Abdominal ultrasonography revealed massive hemoperitoneum. Laboratory tests showed severe anemia with hemoglobin of 4.0 g/dL. Blood product resuscitation was started immediately and emergency laparotomy was performed given the hemodynamic instability. 

More than 4 litres of blood and clots were evacuated. Two major sources of bleeding were finally found: the liver metastasis in segment VI, which displayed a huge increase in size to involve almost the entire right lobe, and the splenic hilum, where splenic vein thrombosis was identified. Splenic vein thrombosis occurred although the patient had taken antithrombotic prophylaxis until postoperative day 28. The first source of bleeding was controlled with bipolar forceps and resorbable hemostatic agents. The second source required splenectomy and resection of the splenic flexure of the colon as well. In the context of damage control surgery, abdominal packing and open abdomen management with a vacuum dressing were performed.

The patient initially required significant amounts of noradrenaline in the intensive care unit (ICU), which was then progressively decreased. Surgical revision two days later showed no further bleeding. Since the patient had improved with a decreasing noradrenaline, terminal colostomy and abdomen closure were accomplished.

**Figure 5:**
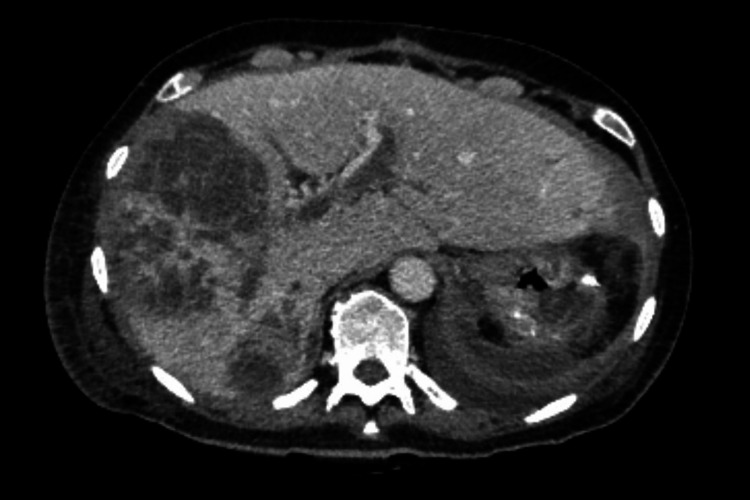
Postoperative abdominal CT scan (venous phase, axial plane): massive progression of liver metastases with neoplastic intrahepatic portal vein thrombosis and necrotic areas one month after surgery. CT: Computed Tomography

**Figure 6:**
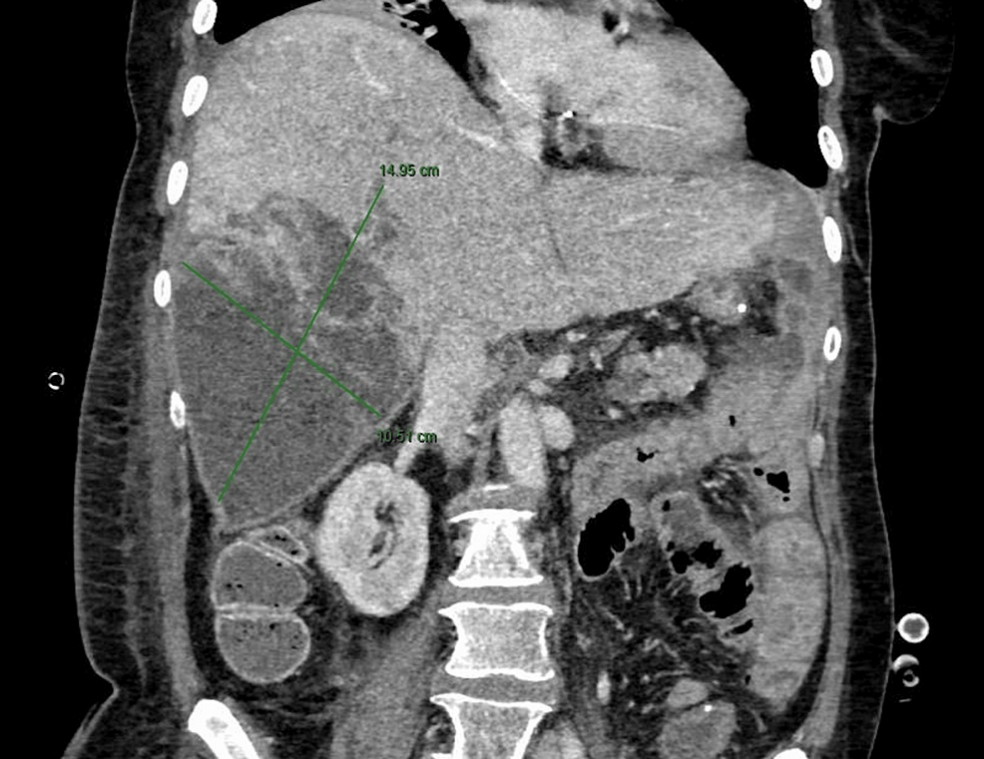
Postoperative abdominal CT scan (venous phase, coronal plane): progression of the liver metastasis in segments VI and VII, with necrotic areas inside. CT: Computed Tomography

The patient gradually recovered and was discharged from ICU two weeks later. However, abdominal CT scan performed 15 days after the surgical revision confirmed the massive progression of liver metastases, with neoplastic infiltration of both the right portal vein and the main portal trunk, being the proximal splenic vein and the spleno-mesenteric-portal confluence patent. Surgical treatment was therefore precluded (Figures 5, 6). Poor clinical condition did not allow any chemotherapy to be considered either. Anticoagulant treatment was started, but the patient's condition gradually deteriorated. The patient was then transferred to the local hospice and received only supportive palliative care. She died one month later.

## Discussion

Glomus tumors are benign mesenchymal neoplasms arising from glomus bodies, which are dermal arteriovenous shunts involved in skin thermoregulation. GTs are unusual, accounting for approximately 2% of all soft tissue tumors. They are composed of modified perivascular cells, similar to those of normal glomus bodies. GTs usually occur in the peripheral soft tissue of the extremities, involving the subungual area of fingers and toes. The majority are benign and malignant GTs represent less than 1% of all GTs [1-3].

GTs rarely affect visceral organs, where glomus bodies are almost absent. Few cases of visceral involvement have been reported in the literature so far, the stomach being the most commonly involved organ. GGTs usually present as masses originating from the submucosa or muscolaris propria and projecting into the lumen or out onto the serosa. Most GGTs are symptomatic with abdominal pain, GI bleeding or perforation, although some cases have been diagnosed incidentally. Among GGTs, malignant variants are exceedingly rare [1-3].

GGTs share many overlapping features with other stromal lesions such as gastrointestinal stromal tumors (GIST), which they are frequently mistaken for, and carcinoid tumors. It is difficult to distinguish these lesions from other gastric submucosal tumors because of the lack of specific clinical, radiological and endoscopic characteristics. Hence, the diagnosis of GGTs is based on pathological and immunohistochemical findings. Endoscopic ultrasonography-guided fine needle aspiration, endoscopic mucosal resection, and endoscopic submucosal dissection are available procedures for histological diagnosis [5].

The presence of nuclear atypia and mitotic activity has been advocated to predict tumor behavior. In particular, according to the 2013 WHO classification, the term malignant GT can be used only in the presence of marked nuclear atypia and mitotic activity; on the other side, in the absence of nuclear atypia, tumors size greater than 2.0 cm and deep location are features of uncertain malignant potential [6].

Despite being malignant or of uncertain malignant potential, GGTs usually do not metastasize. Only a handful of cases of metastatic GGTs have been reported in the literature so far [7-12]. The following reports are those we have identified through PubMed, using specific keywords and/or Medical Subject Heading (MeSH) terms (i.e., “glomus tumor”, “stomach”, “gastric”, “metastases”).

In a series of 52 different GTs published by Folpe et al. in 2001, the only GGT present did show metastatic behavior. Specifically, the patient had an 8.5 cm gastric tumor; he died from liver recurrence three years after gastric surgery [7].

In 2002, Mittenen et al. studied the pathological features of 32 gastrointestinal GTs, of which 31 were GGTs, and concluded that they were histologically and immunohistochemically similar to peripheral GTs. Among the 13 patients with follow-up, only one with an ulcerated 6.5 cm GGT of the antrum developed liver metastases and died 50 months after gastric surgery. Considering the study by Folpe et al., Mittenen et al. assumed that GGTs larger than 5 cm could have a potential malignant behavior [8].

Bray et al. in 2009 reported a skin metastasis in a patient who had undergone subtotal gastrectomy for a presumed GIST six years earlier. Re-evaluation of pathological slides led to a revised diagnosis of primary malignant gastric GT with a single metachronous skin metastasis [9].

Lee et al. also reported two cases of metastatic GGT in 2009. The first patient had a 3 cm GGT of the fundus with right kidney and brain metastases. One month after combined wedge gastric resection and right nephrectomy, he underwent neurosurgery. He died eight months after the initial diagnosis, having developed two metastatic lesions, in the left humeral head and again in the brain. The second patient, on the other hand, was diagnosed with a voluminous 9 cm GGT along the lesser curvature, with multiple synchronous liver metastases and extensive paraaortic lymphadenopathy. After completion of the first chemotherapy cycle, the patient died due to massive bleeding from the GGT [10].

In 2010, Song et al. published the case of a 65-year-old woman suffering from a 3 cm GGT with synchronous kidney, brain and bone metastases. She first underwent wedge resection of the stomach and radical nephrectomy, and then resection of the encephalic metastases and radiotherapy to both the brain and the bone. She was in poor general condition to receive chemotherapy, and she died seven months after diagnosis due to cerebral recurrence [11].

More recently, Bodolan et al. reported the case of an 80-year-old woman with a 7.1 cm mass of the gastric antrum, which was consistent with a GIST on upper endoscopy and CT scan. Only after two endoscopic biopsies a definitive diagnosis of GGT was obtained. Unfortunately, at the time of the planned gastric surgery, multiple hepatic nodules were found; on frozen sections, they displayed the same histomorphology as the GGT. Gastric resection was therefore aborted and palliative chemotherapy was started [12].

Finally, another case of metastatic GGT has been described by Toti et al. A 72-year-old man underwent surgical resection of a 10 cm liver neoplasm, which was consistent with malignant mesenchymal tumor. The following month, a CT scan detected a dishomogeneous 6 cm gastric mass in the greater curvature, confirmed by upper endoscopy. Endoscopic biopsies revealed a mesenchymal tumor. Relaparotomy and wedge resection of the stomach were then performed, together with radiofrequency ablation (RFA) of an additional liver lesion. The pathological findings allowed the diagnosis of malignant GGT; re-assessment of the previous liver slides showed that the liver neoplasm was indeed a GGT metastasis [13]. These reports are all summarized in Table 2.

**Table 2:** Metastatic gastric glomus tumors reported in the literature so far

Author	Year	Age	Sex	Location	Tumor size	Mitoses per high-power field	Atypia	Vascular invasion	Presence of metastases and follow-up
Folpe et al. [7]	2001	69	M	Not known	8.5 cm	3/50	No	Yes	No synchronous metastases. Death from metachronous hepatic metastases three years after gastric surgery.
Miettinen et al. [8]	2002	69	M	Antrum	6.5 cm	1/50	Yes	Yes	No synchronous metastases. Death from metachronous hepatic metastases 50 months after gastric surgery.
Bray et al. [9]	2009	58	M	Antrum	11 cm	>15/50	Yes	Yes	No synchronous metastases. Single metachronous metastasis on the scalp vertex developed six years after gastric surgery.
Lee et al. [10]	2009	65	F	Fundus	3 cm	2/50	Yes	No	Synchronous kidney and brain metastases. Death from humeral and brain recurrence eight months after the diagnosis.
Lee et al. [10]	2009	63	M	Lesser curvature and cardia	9 cm	---	Yes	Yes	Synchronous liver metastases and extensive paraaortic lymphadenopathy. Death from gastric tumour bleeding after the first cycle of chemotherapy.
Song et al. [11]	2010	65	F	Fundus	3 cm	2/50	Yes	Yes	Synchronous kidney, brain, bone metastases. Death from brain recurrence seven months after the diagnosis.
Bodolan et al. [12]	2018	80	F	Antrum	7.1 cm	28/10	Yes	No	Synchronous liver metastases discovered at the time of gastric resection, which was aborted. Palliative chemotherapy was started
Toti et al. [13]	2019	72	M	Greater curvature	6 cm	14/10	Yes	Yes	Two synchronous liver metastases. No data on follow-up.

As demonstrated by the above reports, the pathological diagnosis of GGTs is difficult and sometimes not even obtained on the surgical specimen, but only after the appearance of metastatic localizations. In our case, in particular, the exact diagnosis could not be determined before surgery, although both a gastric and a liver biopsy were available and a second opinion was provided by the regional oncologic hub.

Another similarity with the few known metastatic cases is the size of the primary tumor; indeed, our 12 cm GGT is the largest described so far. This case report, therefore, supports the 2013 WHO hypothesis that, in the absence of marked nuclear atypia, dimensions greater than 2 cm may indicate a malignant potential.

Focusing on secondary localizations from GGTs, this report is the first to describe a dramatic progression of synchronous liver metastases, with the development of neoplastic intrahepatic portal thrombosis also. This happened within a limited time frame, with gastric surgery in between; immunosuppression resulting from this surgery has certainly played an important role in tumor progression.

Wide local excision is considered the treatment of choice for GGTs; in cases of large GGT, when free-margin excision is not feasible, subtotal or total gastrectomy may also be considered [3,12]. Whenever possible, our experience with this patient suggests the simultaneous resection of any synchronous liver metastases, given their potential rapid growth and risk of bleeding. Major liver resections, defined as resections of three or more liver segments according to the Brisbane 2000 terminology [14], are generally not performed during complex gastro-intestinal surgical procedures, due to the high complication rate. Indeed, we also opted for deferred liver surgery. However, considering the rapid progression of liver metastases in our case, we believe that major liver resections might be combined with gastric surgery in case of metastatic GGT, provided that future liver remnant is adequate. Simultaneous treatment with RFA, according to Toti et al.’s experience, remains an option for small metastases when surgical resection is not possible.

## Conclusions

Gastric glomus tumors are rare and usually benign neoplasms, the diagnosis of which is always challenging. Only a few cases of metastatic GGTs have been published in the literature, and those with synchronous metastases have all shown aggressive behavior.

In particular, our report suggests that the resection of any synchronous liver metastases should possibly be performed at the same time as the GGT excision and not at a later stage, given their dramatic progression and risk of bleeding.
